# The Clinical Trials Landscape for Alzheimer's Disease

**DOI:** 10.1111/cns.70492

**Published:** 2025-06-26

**Authors:** Guoping Shen, Xue Xu, Minping Li, Zaiyuan Sun, Linyu Wei, Zhezhi Deng, Zehuang Lin, Jianwen Huang, Weiwei Qi, Jia Xu

**Affiliations:** ^1^ Department of Radiation Oncology The First Affiliated Hospital of Sun Yat‐Sen University Guangzhou Guangdong PR China; ^2^ Department of Neurology The First Affiliated Hospital of Sun Yat‐Sen University Guangzhou Guangdong PR China; ^3^ Department of Emergency The Seventh Affiliated Hospital of Sun Yat‐Sen University Guangzhou Guangdong PR China; ^4^ Department of Emergency The First Affiliated Hospital of Sun Yat‐Sen University Guangzhou Guangdong PR China

**Keywords:** Alzheimer's disease, clinical trials, cognition, dementia, landscape

## Abstract

**Introduction:**

Not only drug and non‐drug development but also non‐therapeutic research in Alzheimer's disease (AD) clinical trials is unclear.

**Methods:**

The participants were AD clinical trials obtained from the clinicaltrials.gov registry. The research objectives and interventions of those trials were analyzed. Bibliometric network analysis of those published articles in PubMed was also conducted.

**Results:**

A total of 1681 clinical trials and 565 corresponding published articles in the past 20 years were included in the analysis. “Safety”, “dose”, “adverse events”, and “biomarker” were the most frequently used words that appeared in the title or abstract of published articles. The top three classes of 362 drugs were anti‐Amyloid, enhancing acetylcholine, neurotransmitter, or targeting its receptor. The physical therapy, diet, and cognitive training ranked as the first three classes of non‐drug therapy. Imaging, risk factors, and molecular biomarkers were the three most abundant categories in non‐therapeutic research, and three fields of prevention, risk factors, and the dental or intestinal microbiome showed an escalated trend (All *P*_trend_ < 0.05).

**Dissusion:**

The results described a comprehensive landscape for the clinical studies of AD. Although most drugs treated AD abortively, the success of lecanemab and decanemab provides confidence for us to further study the pathogenesis of AD and explore new therapeutic targets to develop anti‐AD drugs. Rising non‐drug and non‐therapeutic research will provide more possible methods for the treatment and prevention of AD in thefuture.

## Graphical abstract

The interventions in AD trials were classified into drug, non‐drug therapy, which included 17, and 17 subcategories, respectively; The study endpoints of AD trials were divided into 5 categories, of which the non‐therapeutic studies were further divided into 13 subcategories. The research trends in interventions and study endpoints were described.

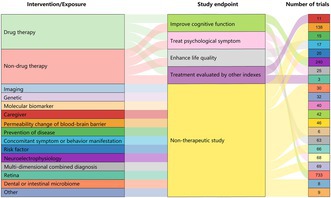

## 1 Background

Alzheimer's disease (AD) is an acquired degeneration disease of the brain characterized by clinical feature of progressive cognition impairment start with recent memory typically. By far, the pathophysiology of AD is thought to be the formation of Amyloid plaques and neurofibrillary tangles (NFTs) and followed by damage and death of neural cells. As the most common cause of dementia, AD is estimated to affect 44 million people world‐widely at present and this number will triple by 2050 as the population ages. The estimated 2021 cost of caring for those with AD and related dementia (ADRD) was $355 billion [1]. Besides the heavy economical burden, how to properly taking care of growing elder population with impaired ability of daily life became a public health crisis. The therapy to prevent, delay the onset, slow the progression, and improve the symptoms of AD is needed urgently.

Clinical trial is the best way to evaluate whether a therapy is safe and effective for AD. Under the great pressure of social and economic issue brought about by AD, many clinical trials have been taken to search for effective drugs and non‐drug treatments. As for drug development, Phase I study involves the First‐in‐Human (FIH) exposure of the drug, and usually enrolls healthy volunteers to collect information of the absorption, distribution, metabolism, excretion, and toxicity of the drug and to find the maximum tolerated dose, dose range for phase II study. Phase II study is taken in a relatively small population of interest, include preclinical AD, prodromal AD and AD dementia. The goal of Phase II is to gain confidence in the treatment, especially to find the target engagement or proof of pharmacology, and to provide information for Phase III trials. The learnings of Phase II are tested in Phase III and, if benefits are confirmed, the agent will be submitted to the FDA for review. Phase IV studies will be carried out after the drug has been approved by the FDA or other regulatory agency and is available on the market. On average, development for an AD treatment requires 13 years and is expected to cost $5.6 billion U.S. dollars. Preclinical evaluation requires approximately 2 years, Phase I averages 2.8 months, Phase II requires 27.7 months, Phase III is typically 50.9 months, and FDA review requires 18 months [2].

We review the clinical trials for AD from the 20th century to December 2023. We present data from analyses of the clinicaltrials.gov registry [3]. Our goal is to provide the trend of diagnostic technology and therapeutic development of AD, that may shed some light on future research in the field of AD prevention and treatment.

## 2 Methods

We used the US National Library of Medicine of the National Institutes of Health (NIH) clinical research registry, clinicaltrials.gov, as the source of information for this review. We also searched in PubMed for articles published about those trials till December 31, 2023 by using NCT numbers, and analyzed the titles and abstracts of these articles using VOSviewer1.6.18. Ethical approval for the study was waived by the Ethics Committee to the First Affiliated Hospital of Sun Yat‐sen University in China.

These clinical trials were classified into 5 categories according to the primary purpose (study endpoint) of the trial: improve cognition, treat psychological symptoms (such as agitation, apathy, depression), enhance quality of life (such as sleep improvement, pain management), treatment evaluated by other indexes (such as blood biochemical indicators), and non‐therapeutic studies.

Based on intervention of trials, the trials were divided into three categories: drug therapy (the intervention of trials is drug), non‐drug therapy (the intervention of trials is non‐drug), and non‐intervention trials whose study objects are various exposures referred to as environmental, behavioral, biological, social, or medical factors. The study endpoints of non‐intervention trials were not to treat disease, also referred to as non‐therapeutic studies (see graphical abstract image).

Non‐therapeutic studies include: study of imaging, genetic, molecular biomarker, caregiver, permeability change of blood–brain barrier, prevention of disease; concomitant symptom or behavior manifestation; risk factor; neuroelectrophysiology; multi‐dimensional combined diagnosis; retina; dental or intestinal microbiome; and others (such as database building, cognitive measurement scale, socioeconomic, and patient management, etc.).

Interventions for these clinical trials are divided into drug and non‐drug. Drugs include the following categories: 1. Enhance acetylcholine; 2. NMDA (N‐methyl‐D‐aspartic acid) receptor related (antagonist or enhancer); 3. Anti‐Amyloid; 4. Anti‐Tau protein; 5. Growth factors (including nerve growth factor, granulocyte colony stimulating factor etc.); 6. Anti‐inflammation/immunoregulation; 7. Vascular protection (blood pressure management, lipid‐lowering, hypoglycemic, anticoagulation); 8. Neurotransmitter or targeted its receptor; 9. Gene and cell therapy (Genes such as NGF (Nerve Growth Factor) and BDNF (Brain‐Derived Neurotrophic Factor) are targeted into brain tissue through viral vectors, or the depleted neuronal circuits are regenerated by autologous or allogeneic stem cells); 10. Sex hormone include estrogen, progesterone, and testosterone; 11. Non‐sex hormone or their regulators, for example, growth hormone, melatonin, orexin, etc.; 12. Herbal or plant extract (see the details in Table S2); 13. Levetiracetam; 14. Targeted therapy of cell signaling pathways (such as Kit, Src and other kinase inhibitors, anti‐aging combination of Dasatinib and Quercetin); 15. Epigenetic therapy (e.g., Vorinostat, Vafidemstat); 16. Calcium regulation; and 17. Others.

Non‐drug include the following categories: 1. Physical therapy; 2. Diet or metabolic nutrition supplement; 3. Exercise; 4. Acupuncture; 5. Art (Music/Garden); 6. Hearing/Visual aids (e.g., wearing hearing‐aid, surgery to remove cataract crystals); 7. Surgery (clearance of neurotoxin by COGNIShunt System and omental transposition Surgery); 8. Cognitive training/Reminiscence therapy (including those with the help of APP (cellphone, iPad or computer application) and virtual reality (VR), etc.); 9. Continuous positive airway pressure (CPAP); 10. Occupational therapy; 11. Radiotherapy; 12. Life or behavioral intervention/management; 13. Immunoadsorption; 14. Auricular point acupressure (APA); 15. Semantic therapy; 16. Compression of vascular beds; and 17. Hyperbaric oxygen therapy.

The difference between Physical therapy and radiotherapy is that radiotherapy refers exclusively to ionizing radiation, whereas physical therapy uses non‐ionizing radiation such as sound, light, electricity, magnetism and heat as interventions, include: Electroconvulsive therapy (ECT), Deep brain stimulation (DBS), Thermal stimulation, theta burst transcranial magnetic stimulation or Transcranial magnetic stimulation (TMS), Transcranial alternating current stimulation (tACS), Transcranial direct current stimulation (tDCS), Transcranial electromagnetic treatment (TEMT), Transcranial photobiomodulation (t‐PBM), Transcranial electrical stimulation (tES), Transcutaneous vagus nerve stimulation (tVNS), and others.

The index date for this review is December 31, 2023, and the tables and text apply to the information available on that date. We include all trials associated with AD and divide them according to start year as studies before 2005 and every 3 years since 2006 to analyze the trend change with time. We analyze information on the trial intervention, phase, type, design, number of subjects planned for enrollment, subject characteristics (e.g., age, gender), fund resource, etc. The trend was tested using the Cochran‐Armitage test with *p* ≤ 0.05 as statistical significance. All the statistical analyses were performed using the R Foundation for Statistical Computing version 3.6.1. The bubble figures were produced using Tableau Software (v2019.4.4).

## 3 Results

### 3.1 General View

#### 3.1.1 Characteristics of AD Trials

Till December 31, 2023, a total of 444,508 clinical trials had been registered in clinicaltrials.gov. Filtered by “Alzheimer” in the title, total 1681 clinical trials were identified Alzheimer's disease research. Key characteristics are listed in table 1. Phase I to II trials accounted for 38.2%; only 11.5% were in phase III and 4.9% were in phase IV. The rest 27.3% trials were not available on this information and 16.0% were observational trials which were not applicable for phase. Most clinical trials include adult and/or older adult, but 6.1% trials include child. Nearly all trials include male and female but still there were 1% trials include only female and 0.5% include only male subjects. The number of enrollments were less than 50 in about 1/3 of all clinical trials, and more than 500 in 13.3% clinical trials. Government only funded 10.8% of all clinical trials while pharmacy industry funded nearly half (44.7%) of all. Most (84.0%) clinical trials were interventional, and randomized study design accounted for 58.9%. The drug therapy and non‐drug therapy accounted for 49.3% and 19.7% in therapeutic studies.

#### 3.1.2 Trends of AD Trials

**FIGURE 1:**
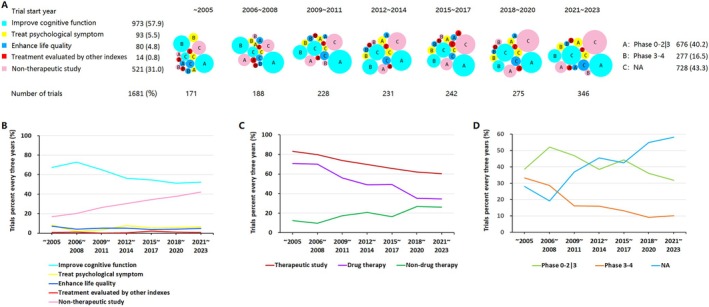
Landscape of AD clinical trials. (A) Distribution of clinical trials according to research purpose, start year and trial phase (the size of the bubble correlated to the counts of trials). (B) The trend of research purpose changing every 3 years since 2006. (C) The trend of intervention changing every 3 years since 2006. (D) The trend of clinical trial phase changing every 3 years since 2006. NA, not available or not applicable.

The number of clinical trials about AD has grown rapidly after 2005 (Figure 1A). Before 2005 there were only 171 clinical trials, but from 2006 to 2008 the number of new clinical trials reached 188 in 3 years, and this number grew to 346 between 2021 and 2023. The five types of trial endpoints of improve cognition, treat psychological symptoms, enhance quality of life, treatment evaluated by other indexes, and non‐therapeutic studies accounted for 57.9%, 5.5%, 4.8%, 0.8%, and 31.0%, respectively.

Comparison of data of every 3 years shows improving cognitive function remained the most important purpose of clinical trials of AD while more and more non‐therapeutic studies were sponsored as time goes by. The number of clinical trials of enhance life quality and treatment of psychological symptoms study almost remained stable during time. Although improving cognitive function is the main research purpose, its proportion decreased while the proportion of non‐therapeutic study increased from 2005 to 2023 (Figure 1B). In trend of research purpose changing every 3 years, *P*_trend_ were < 0.001, 0.406, 0.342, 0.017, and < 0.001 for improve cognitive function, treat psychological symptom, enhance life quality, treatment evaluated by other indexes, and non‐therapeutic study, respectively. When the proportion of non‐therapeutic study increased, the therapeutic study proportion decreased in the past two decades. Two parts of therapeutic study presented opposite trends. The trend of drug therapy trials was a descending line while the trend of non‐drug therapy trials appeared ascending (Figure 1C). In trend of therapeutic study changing every 3 years, *P*_trend_ were < 0.001, < 0.001, and < 0.001 for therapeutic study, drug therapy, and non‐drug therapy, respectively.

Speaking from the perspective of clinical trial phase, early phase studies (Phase 0–2|3) were much more than late phase studies (phase 3–4). In the last 2 decades, the percentage of early phase studies fluctuated, but the percentage of late phase studies decreased by year (Figure 1D). In the trend of clinical trial phase changing every 3 years, *P*_trend_ was < 0.001, < 0.001, and < 0.001 for Phase 0–2|3, Phase 3–4, and NA (not available or not applicable), respectively.

#### 3.1.3 Publications of AD Trials

**FIGURE 2:**
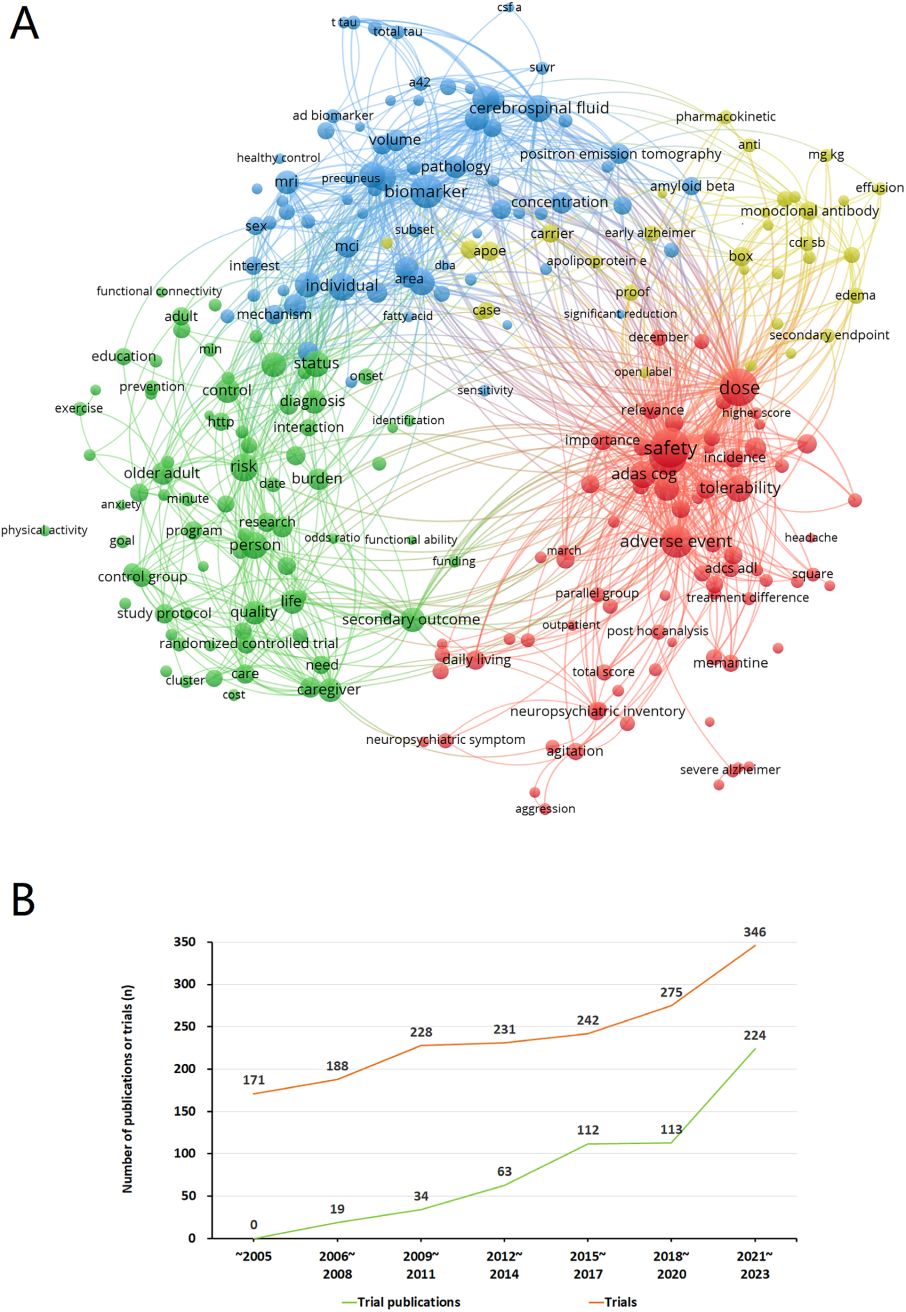
Corresponding AD clinical trial publications. (A) VOS viewer of 565 publications. The size of node represents the counts of keywords in abstracts in 565 publications of AD clinical trials. The more count of one keyword, the bigger of one node. The curve line between two nodes means the frequency of co‐occurrence of two keywords in one abstract. The higher frequency of co‐occurrence of two keywords, the shorter of curve line. Four different colors mean four clusters: Blue nodes clustered AD research directions in tracking pathological proteins; Green nodes clustered the characteristics of population of AD; Red nodes clustered the evaluation dimensions of study endpoint in clinical trials; Yellow nodes clustered the descriptions of drug in trials. (B) The trend of trial publication rises with clinical trial grows.

Total 391 trials have published 565 papers. Among them, there were 240 trials with just one published article. In 829 trials of drug therapy, 331 trials of non‐drug therapy, and 521 trials of non‐therapeutic study, the number of trials that have publication were 232 (28.0%), 73 (22.1%), and 86 (16.5%), respectively. The titles and abstracts of 565 published articles were analyzed. The frequency of key words “safety”, “dose” and “adverse event” are relatively high (Figure 2A), which is consistent with the fact that most AD clinical trials are early phase studies designed to preliminarily explore on drug dosage, safety, and adverse events. With the growth of the trial number, the publication of clinical trial outcomes increased synchronously (Figure 2B).

### 3.2 Clinical Trials of Drugs

**FIGURE 3:**
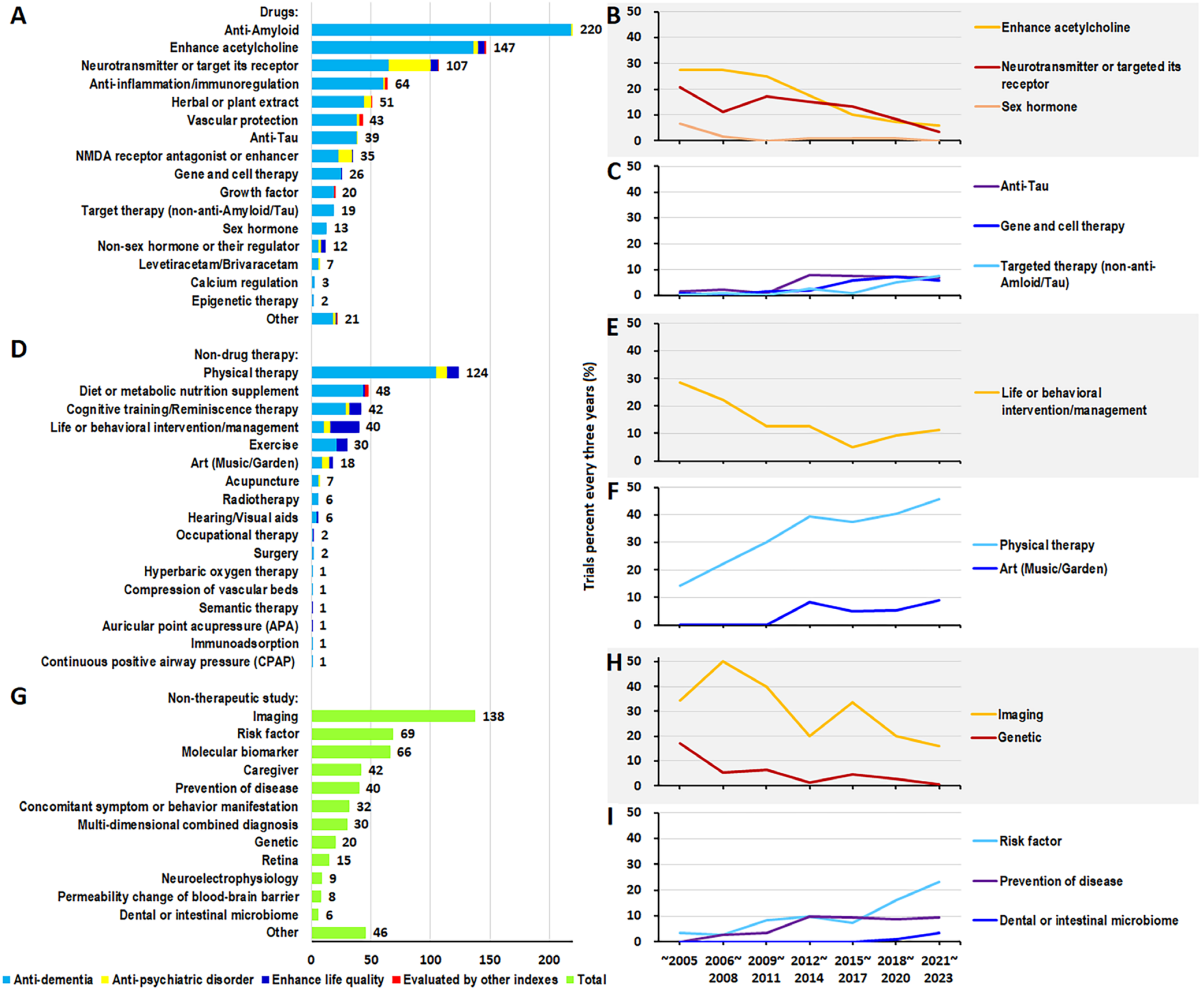
Detail quantity in therapeutic and non‐therapeutic trials. (A) The number of drug clinical trials of different pharmacology mechanism. (B) Decreased trend of clinical trial proportion of enhance acetylcholine, neurotransmitter, and sex hormone. (C) Increased trend of clinical trial proportion of anti‐Tau, gene and cell therapy, and targeted therapy (non‐anti‐Amyloid/Tau). (D) The number of non‐drug clinical trials of different mechanism. (E) Decreased trend of clinical trial proportion of life or behavioral intervention/management. (F) Increased trend of clinical trial proportion of physical therapy and art therapy. (G) The number of non‐therapeutic clinical trials of different aspects. (H) Decreased trend of clinical trial proportion of imaging and genetic study. (I) Increased trend of clinical trial proportion of prevention of disease, risk factor, and dental or intestinal microbiome.

Sorted clinical trial numbers by pharmacology mechanism of 362 drugs from more to less, the clinical trial category were as follow: anti‐Amyloid, enhance acetylcholine, neurotransmitter or targeted its receptor, anti‐inflammation/immunoregulation, herbal or plant extract, vascular protection, anti‐Tau, NMDA receptor antagonist or enhancer, gene and cell therapy, growth factor, targeted therapy (non‐anti‐Amyloid/Tau), sex hormone, non‐sex hormone or their regulator, levetiracetam/brivaracetam, calcium regulation, epigenetic therapy, and others (Figure 3A). There are 220, 147, and 107 drugs in top 3 classes of drugs explored in AD clinical trials respectively. For each mechanism, most trials aimed anti‐dementia, but neurotransmitters and NMDA receptor had a relatively large part of trials aimed anti‐psychiatric disorder. Drugs that enhance acetylcholine, of neurotransmitters and non‐sex hormone had some trials aimed improve quality of life.

Comparison of research period shows a decreased trend of drugs that enhance acetylcholine, neurotransmitters, and sex hormone (Figure 3B), and an increased trend of drugs that anti‐Tau, gene and cell therapy, and targeted therapy (non‐anti‐Amyloid/Tau) (Figure 3C). *P*_trend_ were all < 0.05 for above six categories.

**FIGURE 4:**
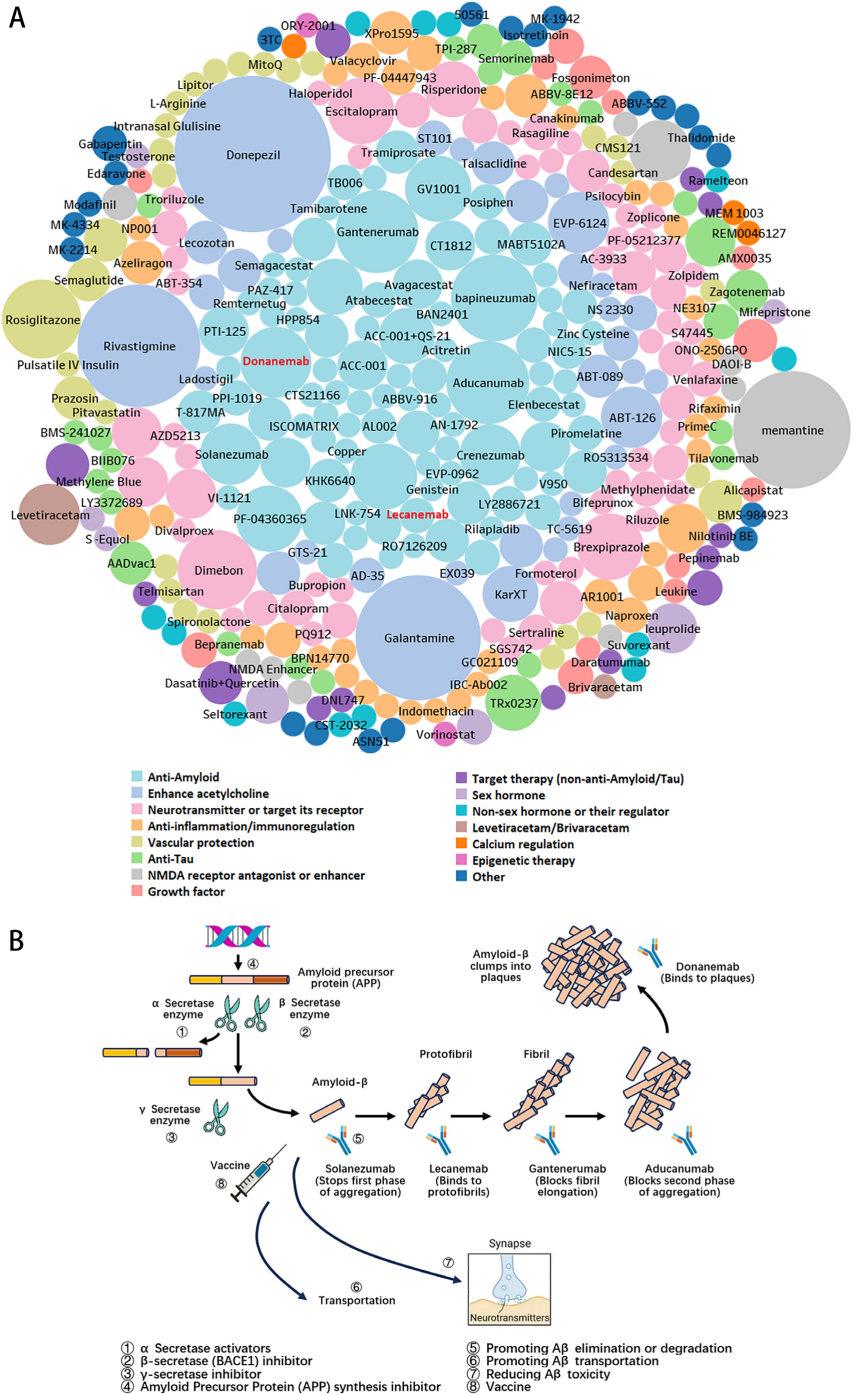
The categories of all drugs in AD clinical trials. (A) The size of the bubble circle represents the number of trials for each drug. Lecanemab and donanemab (red font) have been proved their anti‐dementia effect in recent years. Because of the crowed space, some drug names were not shown. The drug name and corresponding trial counts can be reviewed in Table S1. (B) Eight categories of anti‐amyloid drugs.

All drugs were listed in Figure 4A. Until 2023, donepezil, memantine, galantamine, and rivastigmine were the most explored drugs. Lecanemab and donanemab, which are monoclonal antibodies of beta‐amyloid, are the only two drugs that have been reported to have disease‐modifying effects so far.

### 3.3 Clinical Trials of Non‐Drug Therapy

Sorted clinical trial numbers by mechanism of non‐drugs therapy from more to less, the clinical trial categories were as follows: physical therapy, diet, cognitive training/reminiscence therapy, life or behavioral intervention/management, exercise, art therapy, acupuncture, radiotherapy, hearing/visual aids, occupational therapy, surgery, hyperbaric oxygen therapy, compression of vascular beds, semantic therapy, auricular point acupressure (APA), immunoadsorption, continuous positive airway pressure(CPAP) (Figure 3D). There are 124, 48, and 42 in the top 3 classes of non‐drugs therapy in AD clinical trials. Most of the clinical trials in physical therapy, diet, cognitive training, and exercise therapy aimed at anti‐dementia, but more trials in life or behavioral intervention aimed to improve quality of life. Physical therapy, cognitive training, life or behavioral intervention, and art therapy had some trials aimed at anti‐psychiatric disorders.

During 2005 to 2017 the number of clinical trials on life or behavioral intervention/management decreased, but increased slightly after 2017. However, the number of clinical trials on physical therapy and art therapy grows by year (Figure 3E,F). *P*_trend_ were all < 0.05 for above three categories.

### 3.4 Clinical Trials of Non‐Therapeutic Study

Sorted clinical trial numbers by type of non‐therapeutic study from more to less, the clinical trial category was as follow: imaging, risk factor, molecular biomarker, caregiver, prevention of disease, concomitant symptom or behavior manifestation, multi‐dimensional combined diagnosis, genetic, retina, neuroelectrophysiology, permeability changes of blood–brain barrier, and dental or intestinal microbiome (Figure 3G).

The number of genetic studies has been decreased largely ever since 2005, and during 2021 to 2023 there was only one trial on genetic started. The number of imaging study grown rapidly during 2005 to 2008, but also presented a decreased trend. On the other hand, study of prevention and risk factor grows steadily, and the dental or intestinal microbiome study has gradually attracted researcher's attention in recent 3 years (Figure 3H,I). *P*_trend_ were all < 0.05 for above four categories.

## 4 Discussion

### 4.1 Study Design

The fact that the total number of clinical trials about AD increased over time but the proportion of late phase clinical trials decreased might be related to the disappointing current situation that although a lot of medications or techniques were being evaluated for curing AD, few could pass the early stage of clinical trial due to lack of safety or efficacy. The VOS viewer of 557 AD clinical trial publications also presents keywords “safety”, “dose” and “adverse event” frequently, since most AD clinical trials are early phase studies designed to preliminarily explore drug dosage, safety, and adverse events. Another reason might be the increased endeavor in finding other possibilities of AD pathogenesis other than the beta‐amyloid hypothesis, since the failure of several major phase 3 clinical trials using monoclonal antibodies targeting cerebral beta‐amyloid has prompted skepticism about the Amyloid hypothesis [4].

More than half (57.9%, 962/1661) clinical trials were interventional, trying to find a cure for dementia in AD, and randomized study design accounted for 58.5%. Randomized controlled clinical trials (RCTs) are generally regarded as the gold standard to evaluate the safety and efficacy of a certain therapy. But carrying out high‐quality RCTs of large sample size is difficult because of the limitations of patient recruitment, time costs, the randomization method, and follow‐up, etc. In recent years, how to use real‐world evidence to evaluate the effectiveness and safety has become a hot topic, but it might not be suitable for AD clinical trials, especially for drug development of AD, owing to the potential bias. The correct approach should be scientifically to remove barriers of randomization, such as managing randomization based on scientific principles and using advanced technology in patients enrollment and follow‐up to reduce artificial mistakes and maintain data reliability as much as possible.

About 1/3 of all AD clinical trials were small trials with less than 50 enrollments, but there were also 13.3% large trials with more than 500 enrollments. For drug development, small trials are appropriate for earlier‐phase, which accounts for the majority of current clinical trials of AD, to evaluate dose‐effect and safety. However, small trials are unlikely to be informative in establishing the effectiveness of treatments with modest effects and comparing effective treatments to enable better decisions in practice [5]. So late phase clinical trials usually need large enrollment to get enough evidence on effectiveness.

The fact that pharmacy industry funded nearly half (44.6%) of all AD clinical trials while government only funded 11.0% should be noted. On one hand the rapidly increasing AD population creates a potentially huge market for effective drugs which is attractive absolutely to pharmacy industry and they are capable to afford the high price of drug development. On the other hand, further analysis of trials in each specialty may help elucidate this complex mix of funding, trial size, and location so that policies might be enacted to improve the responsiveness of trials to the needs of public health and the overall research community [5].

Since the major clinical manifestation of AD is the progressive degeneration of cognition, improving cognitive function remains the most important purpose of clinical trials of AD in the past decades, although its proportion decreased from around 70% in 2006 to about 50% in 2023. But the proportion of non‐therapeutic studies increased steadily over time to about 40% in the last 3 years. The reason may be related to the failure of several major phase 3 clinical trials using monoclonal antibodies targeting cerebral beta‐Amyloid. As mentioned before, this failure has prompted skepticism about the Amyloid hypothesis, that a linear cascade is initiated by beta‐Amyloid and leads to dementia, so many fundamental researches turned to study further into the pathophysiology mechanism of AD to find novel therapeutic targets, better biomarkers to early diagnosis of AD, and effective measures to prevent AD.

### 4.2 Mechanism of Drugs in AD Clinical Trials

Anti‐Amyloid, enhance acetylcholine, neurotransmitter or targeted its receptor are the top 3 classes of all mechanisms of drugs in AD clinical trials. But the proportion of the latter two classes decreased over time. On the other hand, disease‐modifying therapy such as anti‐Tau, gene and cell therapy, and targeted therapy (non‐anti‐Amyloid/Tau) gains increased attention and accounts for around 10% of clinical trials each in the last 3 years.

The unsatisfied result of clinical trials targeting beta‐Amyloid in the past encouraged the perfection of the beta‐Amyloid hypothesis and exploration of other mechanisms. A theory of cellular phase consisting of feedback and feedforward responses of astrocytes, microglia, and vasculature has been proposed [6]. No longer viewing beta‐Amyloid, Tau, and inflammation as steps along a sequential pathway but as part of a cellular phase of AD pathogenesis. Dysbiosis of microbes in the intestine may also be associated with AD since the intestinal flora is able to influence the activity of the brain and cause its dysfunctions through the microbiota‐gut‐brain axis (MGBA). The possibility of using antibiotics to prevent AD has also been discussed [7]. Evidence also supports that both humoral and cellular immune responses can be effective in clearing beta‐Amyloid [8]. Humoral mechanisms involve binding specific antibodies to beta‐Amyloid with neutralization of toxicity and/or activation of clearance mechanisms, and T cells might play a central role in non‐antibody mediated clearance of beta‐Amyloid, most probably by stimulating microglial cells. Immunotherapy that induces the appropriate T‐cell subset together with protective microglial cells and beta‐Amyloid‐specific antibody responses is also a promising research direction. As research about innate immunity accumulated, microbe exposure is considered one potential trigger for immune activation and Amyloid accumulation [9]. Also, links between vascular factors and AD have clearly been demonstrated at both the clinical and pathological level [10]. Thus, clinical trials of drugs of different mechanisms were carried out, such as anti‐Tau, regulation of inflammation, immunoregulation, vascular protection, gene therapy, and cell therapy, etc. But, until the phase 3 clinical trials of lecanemab and donanemab, a monoclonal antibodies that specifically bind to beta‐Amyloid, reported positive results in 2023 [11, 12], the doubts about the beta‐Amyloid hypothesis have been eliminated.

The anti‐amyloid drugs we mentioned are actually a group of drugs that interfere with different stages of amyloid generation and reduce the production of amyloid. There were 8 categories of anti‐amyloid drugs as follows (Figure 4B): α‐Secretase activators (e.g., Bryostatin [13]), β‐secretase (BACE1) inhibitors (e.g., Lanabecestat [14], Atabecestat [15], Verubecestat [16], Elenbecestat [16]), γ‐secretase inhibitors (e.g., Begacestat [17], Avagacestat [18], Semagacestat [19]), Amyloid Precursor Protein (APP) synthesis inhibitors (e.g., Posiphen [20]), promoting Aβ elimination or degradation (e.g., TB006 [21], Lecanemab [22], Remternetug [23]), promoting Aβ transportation (e.g., Thiethylperazine [24]), reducing Aβ toxicity (e.g., CT1812 [25], Sumifilam [26]), and vaccine [27, 28, 29] (e.g., ALZ‐101 [30]) (see the details in Table S3). Although anti‐amyloid drugs are the mainstream direction of AD therapy and the drugs of lecanemab and donanemab have shown positive results in treating AD, their limitations are controversial. First, efficacy may only appear in early patients and fail to reverse neuronal damage. Second, ARIA risk (Amyloid‐Related Imaging Abnormalities, ARIA) limits the use of anti‐amyloid drugs [15, 31] in high‐risk groups (e.g., APOE ε4 carriers). And, the high treatment cost may also limit the use of this kind of drugs. Besides, it is currently believed that a single targeting of beta‐amyloid is insufficient to prevent disease progression, and combined targeting of multipathways such as tau pathology and neuroinflammation is required [32].

### 4.3 Non‐Drugs Therapy of AD

Physical therapy, diet, cognitive training and life or behavioral intervention are the main classes of non‐drugs therapy of AD, especially physical therapy accounts for around 1/3 of all non‐drugs clinical trials and its proportion also increased over time. As technology advanced, electrical, magnetic, light, and sound technique were used in physical therapy of AD. For example, near‐infrared (NIR) light treatment appears to be safe and potentially beneficial for AD patients. It improves cognitive function and activities of daily living [33] (ChiCTR2100044344); transcranial magnetic stimulation (TMS) improves cognitive function when using together with cognitive training [34].

Art (music and garden) therapy also holds an increasing proportion from 2008. Art therapy engages attention, provides pleasure, and improves neuropsychiatric symptoms, social behavior, and self‐esteem [35]. Music therapy can reduce cognitive decline, especially in autobiographical and episodic memories, psychomotor speed, executive function domains, and global cognition [36]. Participation in gardening may ameliorate symptoms of depression, anxiety, and stress [37].

### 4.4 Non‐Therapeutic Trials of AD

Non‐therapeutic studies are mainly designed to find novel therapeutic targets, better biomarkers to early identify potential AD populations or possible risk factors or associated factors to prevent the development of AD. In all non‐therapeutic studies, imaging studies take the lead. Between 2006 and 2008, imaging studies accounted for more than 50%, but this phenomenon decreased quickly afterward. Genetic studies were close to 20% back in 2005, but decreased to less than 5% in the recent decade. On the other hand, the proportion of research about the risk and associated factors of AD increased over time, especially in the last 5 years, and the proportion of prevention studies increased steadily, reflecting a focus shift from treatment to prevention. Imaging studies, especially PET‐CT images about beta‐Amyloid, and genetic studies expanded and updated the diagnostic technique of AD but cannot provide effective means to hold back the progression of AD. However, risk factor studies provided modifiable factors to delay or prevent AD. The dental or intestinal microbiome study might open new prospects for the prevention or treatment of AD.

## 5 Conclusions

Considering the input–output ratio, besides keeping efforts in drug development, detection of early biomarkers or early diagnostic strategy as well as definition of risk factors and early intervention with effective physical therapy may treatment and prevention of AD in clinics of real world in future.

## Author Contributions

Conceptualization of ideas: Guoping Shen, Weiwei Qi, and Jia Xu Data. Collection: Guoping Shen, Xue Xu, and Weiwei Qi. Data curation: Minping Li, Zaiyuan Sun, Linyu Wei, Zhezhi Deng, Zehuang Lin, and Jianwen Huang. Data analysis/statistics: Guoping Shen. Data interpretation: Guoping Shen, Xue Xu, Weiwei Qi, and Jia Xu. Figure and table creation: Guoping Shen. Writing of initial draft: Weiwei Qi. Writing final manuscript (review and editing): Guoping Shen, Xue Xu, Weiwei Qi, and Jia Xu. Literature search: Zehuang Lin and Jianwen Huang. Funds collection: Guoping Shen.

## Conflicts of Interest

The authors declare no conflicts of interest.

## Supporting information


Data S1.

## Data Availability

The datasets generated during and/or analyzed during the current study are available from the corresponding author on reasonable request.

## References

[1] J. Cummings , G. Lee , P. Nahed , et al., “Alzheimer's Disease Drug Development Pipeline: 2022,” Alzheimers Dement 8, no. 1 (2022): e12295. 10.1002/trc2.12295 PMC9066743 35516416

[2] J. Cummings , A. Ritter , and K. Zhong , “Clinical Trials for Disease‐Modifying Therapies in Alzheimer's Disease: A Primer, Lessons Learned, and a Blueprint for the Future,” Journal of Alzheimer's Disease 64, no. s1 (2018): S3–S22. 10.3233/JAD-179901 PMC6004914 29562511

[3] US and national institutes of health , “About ClinicalTrials.Gov,” http://clinicaltrialsgov.

[4] C. A. Lane , J. Hardy , and J. M. Schott , “Alzheimer's Disease,” European Journal of Neurology 25, no. 1 (2018): 59–70. 28872215 10.1111/ene.13439

[5] R. M. Califf , D. A. Zarin , J. M. Kramer , R. E. Sherman , L. H. Aberle , and A. Tasneem , “Characteristics of Clinical Trials Registered in ClinicalTrials.Gov, 2007–2010,” Journal of the American Medical Association 307, no. 17 (2012): 1838–1847. 22550198 10.1001/jama.2012.3424 PMC13431740

[6] B. De Strooper and E. Karran , “The Cellular Phase of Alzheimer's Disease,” Cell 164, no. 4 (2016): 603–615. 26871627 10.1016/j.cell.2015.12.056

[7] F. Angelucci , K. Cechova , J. Amlerova , and J. Hort , “Antibiotics, Gut Microbiota, and Alzheimer's Disease,” Journal of Neuroinflammation 16, no. 1 (2019): 108. 31118068 10.1186/s12974-019-1494-4 PMC6530014

[8] H. L. Weiner and D. Frenkel , “Immunology and Immunotherapy of Alzheimer's Disease,” Nature Reviews Immunology 6, no. 5 (2006): 404–416. 10.1038/nri1843 16639431

[9] H. E. Whitson , C. Colton , J. El Khoury , et al., “Infection and Inflammation: New Perspectives on Alzheimer's Disease,” Brain, Behavior, & Immunity Health 22 (2022): 100462. 10.1016/j.bbih.2022.100462 PMC9475126 36118272

[10] J. T. O'Brien and H. S. Markus , “Vascular Risk Factors and Alzheimer's Disease,” BMC Medicine 12 (2014): 218. 25385509 10.1186/s12916-014-0218-y PMC4226870

[11] C. H. van Dyck , C. J. Swanson , P. Aisen , et al., “Lecanemab in Early Alzheimer's Disease,” New England Journal of Medicine 388, no. 1 (2023): 9–21. 36449413 10.1056/NEJMoa2212948

[12] J. R. Sims , J. A. Zimmer , C. D. Evans , et al., “Donanemab in Early Symptomatic Alzheimer Disease: The TRAILBLAZER‐ALZ 2 Randomized Clinical Trial,” Journal of the American Medical Association 330 (2023): 512–527. 37459141 10.1001/jama.2023.13239 PMC10352931

[13] M. R. Farlow , R. E. Thompson , L. J. Wei , et al., “A Randomized, Double‐Blind, Placebo‐Controlled, Phase II Study Assessing Safety, Tolerability, and Efficacy of Bryostatin in the Treatment of Moderately Severe to Severe Alzheimer's Disease,” Journal of Alzheimer's Disease 67, no. 2 (2019): 555–570. 10.3233/JAD-180759 PMC6398557 30530975

[14] S. Patel , A. V. Bansoad , R. Singh , and G. L. Khatik , “BACE1: A Key Regulator in Alzheimer's Disease Progression and Current Development of Its Inhibitors,” Current Neuropharmacology 20, no. 6 (2022): 1174–1193. 34852746 10.2174/1570159X19666211201094031 PMC9886827

[15] R. Sperling , D. Henley , P. S. Aisen , et al., “Findings of Efficacy, Safety, and Biomarker Outcomes of Atabecestat in Preclinical Alzheimer Disease: A Truncated Randomized Phase 2b/3 Clinical Trial,” JAMA Neurology 78, no. 3 (2021): 293–301. 33464300 10.1001/jamaneurol.2020.4857 PMC7816119

[16] C. Sur , J. Kost , D. Scott , et al., “BACE Inhibition Causes Rapid, Regional, and Non‐Progressive Volume Reduction in Alzheimer's Disease Brain,” Brain 143, no. 12 (2020): 3816–3826. 33253354 10.1093/brain/awaa332 PMC8453290

[17] S. C. Mayer , A. F. Kreft , B. Harrison , et al., “Discovery of Begacestat, a Notch‐1‐Sparing Gamma‐Secretase Inhibitor for the Treatment of Alzheimer's Disease,” Journal of Medicinal Chemistry 51, no. 23 (2008): 7348–7351. 19012391 10.1021/jm801252w

[18] V. Coric , S. Salloway , C. H. van Dyck , et al., “Targeting Prodromal Alzheimer Disease With Avagacestat: A Randomized Clinical Trial,” JAMA Neurology 72, no. 11 (2015): 1324–1333. 26414022 10.1001/jamaneurol.2015.0607

[19] M. Holtta , R. A. Dean , E. Siemers , et al., “A Single Dose of the Gamma‐Secretase Inhibitor Semagacestat Alters the Cerebrospinal Fluid Peptidome in Humans,” Alzheimer's Research & Therapy 8, no. 1 (2016): 11. 10.1186/s13195-016-0178-x PMC4780148 26948580

[20] H. H. Feldman , J. L. Cummings , A. L. Boxer , et al., “A Framework for Translating Tauopathy Therapeutics: Drug Discovery to Clinical Trials,” Alzheimer's & Dementia 20, no. 11 (2024): 8129–8152. 10.1002/alz.14250 PMC11567863 39316411

[21] I. Srejovic , D. Selakovic , N. Jovicic , V. Jakovljevic , M. L. Lukic , and G. Rosic , “Galectin‐3: Roles in Neurodevelopment, Neuroinflammation, and Behavior,” Biomolecules 10, no. 5 (2020): 798. 32455781 10.3390/biom10050798 PMC7277476

[22] N. Villain , V. Planche , M. Lilamand , et al., “Lecanemab for Early Alzheimer's Disease: Appropriate Use Recommendations From the French Federation of Memory Clinics,” Journal of Prevention of Alzheimer's Disease 12, no. 4 (2025): 100094. 10.1016/j.tjpad.2025.100094 PMC12184063 40011173

[23] F. Panza , V. Dibello , R. Sardone , et al., “Successes and Failures: The Latest Advances in the Clinical Development of Amyloid‐Beta‐Targeting Monoclonal Antibodies for Treating Alzheimer's Disease,” Expert Opinion on Biological Therapy 25, no. 3 (2025): 275–283. 39908579 10.1080/14712598.2025.2463963

[24] Thiethylperazine , “In: Drugs and Lactation Database (LactMed(R)),” (2006).

[25] C. H. van Dyck , A. P. Mecca , R. S. O'Dell , et al., “A Pilot Study to Evaluate the Effect of CT1812 Treatment on Synaptic Density and Other Biomarkers in Alzheimer's Disease,” Alzheimer's Research & Therapy 16, no. 1 (2024): 20. 10.1186/s13195-024-01382-2 PMC10809445 38273408

[26] H. Y. Wang , E. Cecon , J. Dam , Z. Pei , R. Jockers , and L. H. Burns , “Simufilam Reverses Aberrant Receptor Interactions of Filamin A in Alzheimer's Disease,” International Journal of Molecular Sciences 24, no. 18 (2023): 13927. 37762230 10.3390/ijms241813927 PMC10531384

[27] R. Wu , F. Sun , W. Zhang , J. Ren , and G. H. Liu , “Targeting Aging and Age‐Related Diseases With Vaccines,” Nature Aging 4, no. 4 (2024): 464–482. 38622408 10.1038/s43587-024-00597-0

[28] M. G. Agadjanyan , N. Petrovsky , and A. Ghochikyan , “A Fresh Perspective From Immunologists and Vaccine Researchers: Active Vaccination Strategies to Prevent and Reverse Alzheimer's Disease,” Alzheimer's & Dementia 11, no. 10 (2015): 1246–1259. 10.1016/j.jalz.2015.06.1884 PMC4630107 26192465

[29] P. Kwan , H. Konno , K. Y. Chan , and L. Baum , “Rationale for the Development of an Alzheimer's Disease Vaccine,” Human Vaccines & Immunotherapeutics 16, no. 3 (2020): 645–653. 31526227 10.1080/21645515.2019.1665453 PMC7227628

[30] H. Zetterberg , J. O. Rinne , A. Sandberg , et al., “Phase 1b Trial on the Safety, Tolerability and Immunogenicity Ofanti‐Amyloid Vaccine ALZ‐101 in Subjects With MCI or Mild AD,” Alzheimer's & Dementia 20 (2024): 1–2.

[31] S. Salloway , J. Wojtowicz , N. Voyle , et al., “Amyloid‐Related Imaging Abnormalities (ARIA) in Clinical Trials of Gantenerumab in Early Alzheimer Disease,” JAMA Neurology 82, no. 1 (2025): 19–29. 39556389 10.1001/jamaneurol.2024.3937 PMC11574721

[32] J. Cummings , Y. Zhou , G. Lee , K. Zhong , J. Fonseca , and F. Cheng , “Alzheimer's Disease Drug Development Pipeline: 2023,” Alzheimers Dement 9, no. 2 (2023): e12385. 10.1002/trc2.12385 PMC10210334 37251912

[33] L. Chen , J. Xue , Q. Zhao , et al., “A Pilot Study of Near‐Infrared Light Treatment for Alzheimer's Disease,” Journal of Alzheimer's Disease 91, no. 1 (2023): 191–201. 10.3233/JAD-220866 36373323

[34] M. Sabbagh , C. Sadowsky , B. Tousi , et al., “Effects of a Combined Transcranial Magnetic Stimulation (TMS) and Cognitive Training Intervention in Patients With Alzheimer's Disease,” Alzheimer's & Dementia 16, no. 4 (2020): 641–650. 10.1016/j.jalz.2019.08.197 31879235

[35] B. Chancellor , A. Duncan , and A. Chatterjee , “Art Therapy for Alzheimer's Disease and Other Dementias,” Journal of Alzheimer's Disease 39, no. 1 (2014): 1–11. 10.3233/JAD-131295 24121964

[36] R. Fang , S. Ye , J. Huangfu , and D. P. Calimag , “Music Therapy Is a Potential Intervention for Cognition of Alzheimer's Disease: A Mini‐Review,” Translational Neurodegeneration 6 (2017): 2. 28149509 10.1186/s40035-017-0073-9 PMC5267457

[37] H. E. Ainamani , W. M. Bamwerinde , G. Z. Rukundo , et al., “Participation in Gardening Activity and Its Association With Improved Mental Health Among Family Caregivers of People With Dementia in Rural Uganda,” Preventive Medical Reports 23 (2021): 101412. 10.1016/j.pmedr.2021.101412 PMC8193614 34159048

