# Supplementary material for: The Clinical Trials Landscape for Alzheimer's Disease
Source: CNS Neurosci Ther. 2025 Jun 26;31(6):e70492. doi: 10.1111/cns.70492 (PMC12202464; doi:10.1111/cns.70492)
Supplement: Supplementary file 1 — Data S1. [file CNS-31-e70492-s001.zip › Supplementary materials.docx]

Supplementary Table 1. Drug name and corresponding trial counts.

| **No.** | **Drugs** | **Trial Counts** | **Class** |
| --- | --- | --- | --- |
| 1 | 50561 | 1 | C15 |
| 2 | 3TC | 1 | C15 |
| 3 | AAB-001 | 1 | C01 |
| 4 | AAB-003 | 2 | C01 |
| 5 | AADvac1 | 3 | C06 |
| 6 | ABBV-552 | 1 | C15 |
| 7 | ABBV-8E12 | 1 | C06 |
| 8 | ABBV-916 | 1 | C01 |
| 9 | ABT-089 | 3 | C02 |
| 10 | ABT-126 | 6 | C02 |
| 11 | ABT-288 | 1 | C03 |
| 12 | ABT-354 | 1 | C03 |
| 13 | ABT-384 | 1 | C11 |
| 14 | ABT-957 | 1 | C08 |
| 15 | ABvac40 | 1 | C01 |
| 16 | AC-3933 | 1 | C03 |
| 17 | ACC-001 | 4 | C01 |
| 18 | ACC-001+QS-21 | 5 | C01 |
| 19 | Acetaminophen | 1 | C04 |
| 20 | ACI-24.060 | 1 | C15 |
| 21 | Acitretin | 1 | C01 |
| 22 | ACP-204 | 1 | C15 |
| 23 | AD-35 | 2 | C02 |
| 24 | Aducanumab | 11 | C01 |
| 25 | Affitope AD01 | 1 | C01 |
| 26 | Affitope AD02 | 3 | C01 |
| 27 | AGN-242071 | 1 | C02 |
| 28 | AL001 | 1 | C06 |
| 29 | AL002 | 2 | C01 |
| 30 | Alicapistat | 1 | C08 |
| 31 | Allopregnanolone | 3 | C03 |
| 32 | ALZ-101 | 1 | C01 |
| 33 | ALZT-OP1 | 2 | C04 |
| 34 | Ampalex | 1 | C03 |
| 35 | AMX0035 | 1 | C08 |
| 36 | AN-1792 | 1 | C01 |
| 37 | ANAVEX2-73 | 3 | C08 |
| 38 | Anti-oxidant | 1 | C08 |
| 39 | APH-1105 | 1 | C01 |
| 40 | AR1001 | 2 | C04 |
| 41 | Aripiprazole | 4 | C03 |
| 42 | ASN51 | 1 | C15 |
| 43 | ASP0777 | 1 | C01 |
| 44 | Atabecestat | 2 | C01 |
| 45 | Atomoxetine | 1 | C03 |
| 46 | Avagacestat | 4 | C01 |
| 47 | AVP-786 | 6 | C07 |
| 48 | AVP-923 | 1 | C07 |
| 49 | AXS-05 | 2 | C07 |
| 50 | AZD1446 | 2 | C02 |
| 51 | AZD3480 | 2 | C02 |
| 52 | AZD5213 | 1 | C03 |
| 53 | Azeliragon | 3 | C04 |
| 54 | BAN2401 | 1 | C01 |
| 55 | bapineuzumab | 12 | C01 |
| 56 | Baricitinib | 1 | C04 |
| 57 | BCG Vaccination | 1 | C15 |
| 58 | Begacestat | 1 | C01 |
| 59 | Bepranemab | 1 | C06 |
| 60 | Bexarotene | 1 | C01 |
| 61 | BI 409306 | 3 | C01 |
| 62 | BI 425809 | 1 | C03 |
| 63 | Bifeprunox | 1 | C03 |
| 64 | BIIB076 | 1 | C06 |
| 65 | BIIB080 | 1 | C06 |
| 66 | BMS-241027 | 1 | C06 |
| 67 | BMS-984923 | 1 | C15 |
| 68 | BPDO-1603 | 1 | C15 |
| 69 | BPN14770 | 1 | C04 |
| 70 | Brexpiprazole | 7 | C03 |
| 71 | Brivaracetam | 1 | C12 |
| 72 | Bromocriptine | 1 | C03 |
| 73 | Bryostatin | 5 | C01 |
| 74 | Bumetanide | 1 | C01 |
| 75 | Bupropion | 1 | C03 |
| 76 | CAD106 | 7 | C01 |
| 77 | Caffeine | 1 | C06 |
| 78 | Canakinumab | 1 | C04 |
| 79 | Candesartan | 1 | C05 |
| 80 | Carvedilol | 1 | C05 |
| 81 | Celecoxib | 2 | C04 |
| 82 | Choline Alfoscerate | 3 | C02 |
| 83 | Cholinesterase Inhibitors | 3 | C02 |
| 84 | Chromium Chloride | 1 | C05 |
| 85 | Circadin | 1 | C01 |
| 86 | Citalopram | 2 | C03 |
| 87 | CMS121 | 1 | C05 |
| 88 | CNP520 | 1 | C01 |
| 89 | Copper | 1 | C01 |
| 90 | COR388 | 1 | C04 |
| 91 | CPC-201 | 3 | C02 |
| 92 | Crenezumab | 6 | C01 |
| 93 | CST-107 | 1 | C11 |
| 94 | CST-2032 | 1 | C11 |
| 95 | CT1812 | 4 | C01 |
| 96 | CTS21166 | 1 | C01 |
| 97 | CY6463 | 1 | C09 |
| 98 | Cyclophosphamate | 1 | C04 |
| 99 | Dabigatran | 1 | C05 |
| 100 | DAOI-B | 1 | C07 |
| 101 | Dapagliflozin | 1 | C05 |
| 102 | Daratumumab | 1 | C09 |
| 103 | Daridorexant | 1 | C11 |
| 104 | Dasatinib+Quercetin | 3 | C09 |
| 105 | Dexmedetomidine | 1 | C11 |
| 106 | Dextroamphetamine | 1 | C03 |
| 107 | Dimebon | 10 | C03 |
| 108 | Divalproex | 1 | C03 |
| 109 | Divalproex+Quetiapine | 1 | C03 |
| 110 | DL-3-n-butylphthalide(NBP) | 1 | C07 |
| 111 | DNL747 | 1 | C09 |
| 112 | Donanemab | 8 | C01 |
| 113 | Donepezil | 39 | C02 |
| 114 | Doxycycline+Rifampicin | 3 | C04 |
| 115 | E2609 | 2 | C01 |
| 116 | E2814 | 1 | C06 |
| 117 | Edaravone | 1 | C15 |
| 118 | EHT 0202 | 1 | C01 |
| 119 | Elenbecestat | 2 | C01 |
| 120 | ELND005 | 4 | C01 |
| 121 | Epigallocatechin-Gallate | 1 | C01 |
| 122 | Escitalopram | 7 | C03 |
| 123 | Estrogen | 3 | C10 |
| 124 | Etanercept | 4 | C04 |
| 125 | EVP-0962 | 1 | C01 |
| 126 | EVP-6124 | 6 | C02 |
| 127 | EX039 | 1 | C02 |
| 128 | Exendin-4 | 1 | C05 |
| 129 | FK962 | 1 | C11 |
| 130 | Formoterol | 1 | C03 |
| 131 | Fosgonimeton | 3 | C08 |
| 132 | Gabapentin | 2 | C15 |
| 133 | Galantamine | 25 | C02 |
| 134 | Gantenerumab | 11 | C01 |
| 135 | GC021109 | 1 | C04 |
| 136 | G-CSF | 2 | C08 |
| 137 | Genistein | 1 | C01 |
| 138 | Gosuranemab | 1 | C06 |
| 139 | GSK239512 | 2 | C03 |
| 140 | GSK4527226 | 1 | C09 |
| 141 | GSK933776 | 2 | C01 |
| 142 | GTS-21 | 1 | C02 |
| 143 | GV1001 | 3 | C01 |
| 144 | GV1001 | 4 | C01 |
| 145 | Haloperidol | 1 | C03 |
| 146 | HF0220 | 1 | C15 |
| 147 | HPP854 | 1 | C01 |
| 148 | Huperzine A | 3 | C02 |
| 149 | Hydralazine | 1 | C09 |
| 150 | Hydroxypropyl Beta Cyclodextrin | 1 | C05 |
| 151 | IBC-Ab002 | 1 | C04 |
| 152 | Ibuprofen | 1 | C04 |
| 153 | ID1201 | 1 | C01 |
| 154 | Idalopirdine | 4 | C03 |
| 155 | Indomethacin | 1 | C04 |
| 156 | Insulin | 1 | C05 |
| 157 | Intepirdine | 2 | C03 |
| 158 | Interferon beta-1a | 1 | C04 |
| 159 | Intranasal Glulisine | 1 | C05 |
| 160 | ISCOMATRIX | 1 | C01 |
| 161 | Isotretinoin | 1 | C15 |
| 162 | ITI-007 | 1 | C03 |
| 163 | JNJ-40346527 | 1 | C08 |
| 164 | JNJ-54861911 | 3 | C01 |
| 165 | JNJ-63733657 | 2 | C06 |
| 166 | KarXT | 2 | C02 |
| 167 | KarXT | 3 | C02 |
| 168 | KHK6640 | 3 | C01 |
| 169 | Ladostigil | 1 | C02 |
| 170 | Lanabecestat | 4 | C01 |
| 171 | L-Arginine | 1 | C05 |
| 172 | Lecanemab | 2 | C01 |
| 173 | Lecozotan | 3 | C02 |
| 174 | Lemborexant | 1 | C11 |
| 175 | Leukine | 1 | C08 |
| 176 | leuprolide | 4 | C10 |
| 177 | Levetiracetam | 6 | C12 |
| 178 | Lipitor | 1 | C05 |
| 179 | Liraglutide | 1 | C05 |
| 180 | lithium | 4 | C06 |
| 181 | LNK-754 | 1 | C01 |
| 182 | Lornoxicam | 1 | C04 |
| 183 | Lovastatin | 1 | C05 |
| 184 | Lu AE58054 | 1 | C03 |
| 185 | Lu AF20513 | 3 | C01 |
| 186 | Lu AF87908 | 1 | C06 |
| 187 | LY2062430 | 3 | C01 |
| 188 | LY2599666 | 1 | C01 |
| 189 | LY2886721 | 2 | C01 |
| 190 | LY3202626 | 2 | C01 |
| 191 | LY3372689 | 1 | C06 |
| 192 | LY3372993 | 2 | C02 |
| 193 | LY450139 | 3 | C01 |
| 194 | LY451395 | 2 | C03 |
| 195 | MABT5102A | 3 | C01 |
| 196 | Magnesium Sulfate | 1 | C01 |
| 197 | Masitinib | 3 | C09 |
| 198 | Masupirdine | 1 | C03 |
| 199 | MEDI1814 | 1 | C01 |
| 200 | Melatonin | 1 | C01 |
| 201 | MEM 1003 | 1 | C13 |
| 202 | MEM 3454 | 1 | C03 |
| 203 | memantine | 22 | C07 |
| 204 | Methylene Blue | 1 | C06 |
| 205 | Methylphenidate | 2 | C03 |
| 206 | Mifepristone | 1 | C10 |
| 207 | Minocycline | 1 | C04 |
| 208 | Mirtazapine | 2 | C03 |
| 209 | MitoQ | 1 | C05 |
| 210 | MK-0249 | 2 | C03 |
| 211 | MK-0677 | 1 | C11 |
| 212 | MK-0952 | 1 | C04 |
| 213 | MK-1942 | 1 | C15 |
| 214 | MK-2214 | 1 | C15 |
| 215 | MK-4334 | 1 | C15 |
| 216 | MK-7622 | 1 | C02 |
| 217 | MK-8189 | 1 | C04 |
| 218 | MK-8931 | 1 | C01 |
| 219 | Modafinil | 1 | C15 |
| 220 | Montelukast | 2 | C04 |
| 221 | MPC-7869 | 3 | C01 |
| 222 | MSDC-0160 | 1 | C05 |
| 223 | MT-4666 | 1 | C02 |
| 224 | MW150 | 1 | C09 |
| 225 | Naproxen | 2 | C04 |
| 226 | NE3107 | 1 | C04 |
| 227 | Nefiracetam | 1 | C02 |
| 228 | Neflamapimod | 2 | C09 |
| 229 | Neramexane | 1 | C07 |
| 230 | Nerve Growth Factor | 1 | C08 |
| 231 | NIC5-15 | 2 | C01 |
| 232 | Nilotinib | 1 | C01 |
| 233 | Nilotinib BE | 1 | C09 |
| 234 | Nilvadipine | 1 | C05 |
| 235 | NIO752 | 1 | C15 |
| 236 | NMDA Enhancer | 1 | C07 |
| 237 | NP001 | 1 | C04 |
| 238 | NP031112 | 1 | C06 |
| 239 | NPT088 | 1 | C01 |
| 240 | NS 2330 | 1 | C02 |
| 241 | Nuedexta | 1 | C07 |
| 242 | Obicetrapib | 1 | C05 |
| 243 | Octohydroaminoacridine Succinate | 2 | C02 |
| 244 | Olanzapine | 1 | C03 |
| 245 | ONO-2506PO | 1 | C04 |
| 246 | ORM-12741 | 2 | C03 |
| 247 | ORY-2001 | 1 | C14 |
| 248 | Paliroden | 2 | C08 |
| 249 | PAZ-417 | 1 | C01 |
| 250 | PBT2 | 1 | C01 |
| 251 | Pepinemab | 1 | C09 |
| 252 | PF-03654746 | 1 | C03 |
| 253 | PF-04360365 | 7 | C01 |
| 254 | PF-04447943 | 2 | C04 |
| 255 | PF-04494700 | 2 | C04 |
| 256 | PF-05212377 | 1 | C03 |
| 257 | Pimavanserin | 3 | C03 |
| 258 | Pioglitazone | 3 | C05 |
| 259 | Piromelatine | 2 | C01 |
| 260 | Pitavastatin | 1 | C05 |
| 261 | Posiphen | 2 | C01 |
| 262 | PPI-1019 | 2 | C01 |
| 263 | PQ912 | 1 | C03 |
| 264 | Prazosin | 3 | C05 |
| 265 | PRI-002 | 1 | C01 |
| 266 | PrimeC | 1 | C04 |
| 267 | PRX-03140 | 3 | C03 |
| 268 | Psilocybin | 1 | C03 |
| 269 | PTI-125 | 4 | C01 |
| 270 | Pulsatile IV Insulin | 1 | C05 |
| 271 | PXT00864 | 1 | C03 |
| 272 | PYM50028 | 1 | C08 |
| 273 | Quetiapine | 2 | C03 |
| 274 | Raloxifene | 2 | C10 |
| 275 | Ramelteon | 1 | C11 |
| 276 | Rapamycin | 1 | C04 |
| 277 | Rasagiline | 2 | C03 |
| 278 | REM0046127 | 1 | C13 |
| 279 | Remternetug | 1 | C01 |
| 280 | Rifaximin | 1 | C04 |
| 281 | Rilapladib | 1 | C01 |
| 282 | Riluzole | 1 | C03 |
| 283 | Risperidone | 5 | C03 |
| 284 | Rivastigmine | 24 | C02 |
| 285 | RO4602522 | 2 | C01 |
| 286 | RO5313534 | 1 | C02 |
| 287 | RO7105705 | 1 | C06 |
| 288 | RO7126209 | 1 | C01 |
| 289 | Rosiglitazone | 10 | C05 |
| 290 | Rotigotine | 1 | C03 |
| 291 | R-pramipexole | 1 | C03 |
| 292 | S -Equol | 1 | C10 |
| 293 | S47445 | 1 | C03 |
| 294 | SAGE-718 | 1 | C07 |
| 295 | Salsalate | 1 | C04 |
| 296 | SAM-531 | 2 | C03 |
| 297 | SAR110894 | 1 | C03 |
| 298 | SAR228810 | 1 | C01 |
| 299 | Saracatinib | 2 | C09 |
| 300 | Sargramostim | 2 | C08 |
| 301 | SB-742457 | 4 | C03 |
| 302 | Seltorexant | 1 | C11 |
| 303 | Semagacestat | 1 | C01 |
| 304 | Semaglutide | 3 | C05 |
| 305 | Semorinemab | 2 | C06 |
| 306 | Senicapoc | 1 | C13 |
| 307 | S-equol | 1 | C10 |
| 308 | Sertraline | 2 | C03 |
| 309 | SGS742 | 1 | C03 |
| 310 | SHR-1707 | 1 | C01 |
| 311 | simufilam | 2 | C01 |
| 312 | Simvastatin | 3 | C05 |
| 313 | Sirolimus | 1 | C09 |
| 314 | SNP318 | 1 | C15 |
| 315 | Solanezumab | 6 | C01 |
| 316 | Solifenacin | 1 | C02 |
| 317 | Spironolactone | 1 | C05 |
| 318 | SSR180711C | 1 | C02 |
| 319 | ST101 | 2 | C02 |
| 320 | SUVN-502 | 1 | C03 |
| 321 | Suvorexant | 1 | C11 |
| 322 | T3D-959 | 1 | C05 |
| 323 | T-817MA | 3 | C01 |
| 324 | Tacrolimus | 1 | C04 |
| 325 | TAK-071 | 1 | C02 |
| 326 | Talsaclidine | 3 | C02 |
| 327 | Tamibarotene | 1 | C01 |
| 328 | Tandospirone | 1 | C03 |
| 329 | TB006 | 2 | C01 |
| 330 | TC-5619 | 1 | C02 |
| 331 | Telmisartan | 1 | C05 |
| 332 | Testosterone | 1 | C10 |
| 333 | Tetrahydrobiopterin | 1 | C05 |
| 334 | Thalidomide | 1 | C15 |
| 335 | Thiethylperazine | 1 | C01 |
| 336 | Tideglusib | 1 | C06 |
| 337 | Tilavonemab | 1 | C06 |
| 338 | TPI-287 | 1 | C06 |
| 339 | Tramiprosate | 3 | C01 |
| 340 | Trazodone | 2 | C03 |
| 341 | Trehalose | 1 | C15 |
| 342 | Troriluzole | 1 | C03 |
| 343 | TRx0014 | 2 | C06 |
| 344 | TRx0237 | 5 | C06 |
| 345 | TTP4000 | 1 | C04 |
| 346 | UB-311 Vaccine | 3 | C01 |
| 347 | Udenafil | 1 | C04 |
| 348 | UE2343 | 1 | C11 |
| 349 | V950 | 1 | C01 |
| 350 | Valacyclovir | 2 | C04 |
| 351 | Varenicline | 1 | C02 |
| 352 | Venlafaxine | 2 | C03 |
| 353 | Verubecestat | 2 | C01 |
| 354 | VI-1121 | 1 | C01 |
| 355 | Vorinostat | 1 | C14 |
| 356 | VX-745 | 2 | C01 |
| 357 | Xaliproden | 2 | C03 |
| 358 | XPro1595 | 3 | C04 |
| 359 | Zagotenemab | 3 | C06 |
| 360 | Zinc Cysteine | 1 | C01 |
| 361 | Zolpidem | 1 | C03 |
| 362 | Zoplicone | 1 | C03 |
| Note:  C01, Anti-Amyloid  C02, Enhance acetylcholine  C03, Neurotransmitter or target its receptor  C04, Anti-inflammation/immunoregulation  C05, Vascular protection  C06, Anti-Tau  C07, NMDA receptor antagonist or enhancer  C08, Growth factor  C09, Target therapy (non-anti-Amyloid/Tau)  C10, Sex hormone  C11, Non-sex hormone or their regulator  C12, Levetiracetam/Brivaracetam  C13, Calcium regulation  C14, Epigenetic therapy  C15, Other | | | |

Supplementary Table 2. The detail of 31 substances of herbal or plant extract.

|  | Herbal or plant extract | Explain | Therapeutic purpose | Trial counts | NCT number |
| --- | --- | --- | --- | --- | --- |
| 1 | EGb 761,  Curcumin and Ginkgo | A Ginkgo biloba extract. | Improve cognition | 4 | NCT00276510  NCT00500500  NCT03090516  NCT00164749 |
| 2 | Curcumin | Curcumin | Improve cognition | 2 | NCT00099710  NCT01001637 |
| 3 | Panax Ginseng | A traditional Chinese herbal medicine | Improve cognition | 1 | NCT00391833 |
| 4 | Isoflavone | One of a class of phytoestrogens, found in soya beans. | Improve cognition | 1 | NCT00205179 |
| 5 | DCB-AD1 | A traditional Chinese herbal medicine comes from the root of Polygonum Multiflorum. | Improve cognition | 1 | NCT00154635 |
| 6 | Sage | Salvia officinalis, a traditional Chinese herbal medicine | Improve cognition | 1 | NCT00110552 |
| 7 | SK-PC-B70M | A traditional Chinese herbal medicine comes from the root of Chinese pulsatilla. | Improve cognition | 2 | NCT00443417  NCT01249196 |
| 8 | ZT-1 | Extract from plants, the detail didn’t disclose. | Improve cognition | 1 | NCT00423228 |
| 9 | lutein | lutein | Whether lutein supplementation helps to reduce oxidative damage from free radicals in AD patients. | 1 | NCT00596024 |
| 10 | INM-176 | INM-176 is a standardized ethanolic extract of Angelica gigas Nakai that has been traditionally used in herbal medicine in China, Japan, and Korea | Improve cognition | 1 | NCT01245530 |
| 11 | Resveratrol | Resveratrol is a polyphenol found naturally in red grapes, peanuts, and many other plant species. | Improve cognition | 3 | NCT00678431  NCT00743743  NCT01504854 |
| 12 | MLC601 | A traditional Chinese herbal medicine, the detail didn’t disclose. | Improve cognition | 1 | NCT01696123 |
| 13 | PM012 | A decoction consisting of several herbs including Rehmanniae Radix Preparata. | Improve cognition | 1 | NCT01715350 |
| 14 | RPh201 | A mastic gum extract | Improve cognition | 2 | NCT01513967  NCT03462121 |
| 15 | 8 Component Botanical Supplement | The detail didn’t disclose. | Improve cognition | 1 | NCT03611439 |
| 16 | Grape Seed Extract | Grape Seed Extract | Improve cognition | 1 | NCT02033941 |
| 17 | DHP1401 | An ethanol extract of the seed of Z. jujuba var. spinosa. | Improve cognition | 1 | NCT03055741 |
| 18 | MLC901 | A simplified formula of MLC601, containing only the 9 herbal components, the detail didn’t disclose. | Improve cognition | 1 | NCT03038035 |
| 19 | GRAPE granules | GRAPE granules was mainly consisted of herbal medicines: Ren Shen (Ginseng, 10g/d), Di Huang (Rehmannia glutinosa, 30g/d), Shi Cangpu (Acorus tatarinowii, 10g/d), Yuan Zhi (Polygala tenuifolia, 10g/d), Yin Yanghuo (Epimedium brevicornu, 10g/d), Shan Zhuyu (Cornus officinalis, 10g/d), Rou Congrong (Cistanche deserticola, 10g/d), Yu Jin (Curcuma aromatica, 10g/d), Dan Shen (Salvia miltiorrhiza, 10g/d), Tian Ma (Angelica sinensis, 10g/d), Tian ma (Gastrodia elata, 10g/d), and Huang Lian (Berberine, 10g/d). | Improve cognition | 1 | NCT03221894 |
| 20 | Cannabidiol, AVE1625,  Nabilone  Dronabinol | Cannabidiol (CBD) is one of at least 85 active cannabinoids identified in cannabis. It is a major phytocannabinoid, accounting for up to 40% of the plant's extract.  AVE1625, a cannabinoid CB1 receptor antagonist.  Nabilone is an analogue of ∆9-tetrahydrocannabinol (THC).  Dronabinol is an orally active cannabinoid. | Treat agitation  Improve cognition | 6 | NCT00380302  NCT04075435  NCT04436081  NCT05822362  NCT02351882  NCT02792257 |
| 21 | Polysaccharide (BAC) | Polysaccharide (BAC) extracted from soybean. | Improve cognition | 2 | NCT02886494  NCT02467413 |
| 22 | VGH-AD1 | A traditional Chinese herbal medicine, the detail didn’t disclose. | Improve cognition | 1 | NCT04249869 |
| 23 | Mycose | Mycose is trehalose. | Improve cognition | 1 | NCT04663854 |
| 24 | Sulforaphane | Sulforaphane is a molecule within the isothiocyanate group of organosulfur compounds. It is obtained from cruciferous vegetables such as broccoli, Brussels sprouts or cabbages. | Improve cognition | 1 | NCT04213391 |
| 25 | GV-971,  Memantine and Oligomannate | GV-971 is Oligomannate. | Improve cognition | 6 | NCT01453569  NCT02293915  NCT04520412  NCT05114499  NCT05908695  NCT05430867 |
| 26 | IGC-AD1 | One of the ingredients of cannabis. | Treat agitation | 2 | NCT04749563  NCT05543681 |
| 27 | SCI -110 | SCI -110 is a combination of dronabinol, and palmitoylethanolamide. | Treat agitation | 1 | NCT05239390 |
| 28 | Yangxue Qingnao Pills | A traditional Chinese herbal medicine, the detail didn’t disclose. | Improve cognition | 1 | NCT04780399 |
| 29 | Blueberry | Blueberry. | Improve cognition | 1 | NCT05172128 |
| 30 | Chinese Traditional Medicine "Smart Soup" | A traditional Chinese herbal medicine., the detail didn’t disclose. | Improve cognition | 1 | NCT05538507 |
| 31 | Wei Li Bai capsules | Wei Li Bai Capsule is composed of sodium ferulate tablets, L-rhamnose and aspicin. | Improve cognition | 1 | NCT05670912 |

Supplementary Table 3. Eight categories of anti-amyloid drugs.

|  | Categories | Mechanism | Representative drugs |
| --- | --- | --- | --- |
| 1 | α Secretase activators | Increases the cleavage of amyloid precursor protein (APP) within the Aβ domain (between Lys16 and Leu17 residues), generating a soluble extracellular fragment, sAPPα, and a membrane-bound C83 fragment. This cleavage prevents the generation of Aβ while enabling sAPPα to exert neuroprotective effects | Bryostatin[13] |
| 2 | β-secretase (BACE1) inhibitor | Blocks BACE1 activity and decreases the production of Aβ | Lanabecestat[14]  Atabecestat[15]  Verubecestat[16]  Elenbecestat[16] |
| 3 | γ-secretase inhibitor | Blocks γ-secreatase activity and decreases the production of Aβ | Begacestat[17]  Avagacestat[18]  Semagacestat[19] |
| 4 | Amyloid Precursor Protein (APP) synthesis inhibitor | Suppresses APP translation, Inhibits the synthesis of APP, Tau and α-Synuclein | Posiphen(ANVS-401) [20] |
| 5 | Promoting Aβ elimination or degradation | Various monoclonal antibodies targeting different stages of Aβ production（first phase aggregation，fibril elongation, second phase aggregation, plaque formation) reduces Aβ plaque formation and promotes Aβ degradation and elimination | TB006 (Targeting Galectin-3) [21]  Lecanemab (BAN2401, targeting protofibrils) [22]  Remternetug (LY3372993, targeting N3pG modulated Aβ plaques) [23] |
| 6 | Promoting Aβ transportation | Enhances clearance of Aβ by up-regulating the membrane transport protein ABCC1 (ATP Binding Cassette Subfamily C Member 1) | Thiethylperazine (TEP) [24] |
| 7 | Reducing Aβ toxicity | prevents and displaces binding of Aβ oligomers, promotes the clearance of Aβ oligomers and blocks α7nAChR and TLR4 pathways of activating Aβ42 signal transduction, so reduces excessive inflammatory cytokines and neuroinflammation | CT1812 (sigma-2 receptor ligand) [25]  Sumifilam (PTI-125, Filamin A binder) [26] |
| 8 | Vaccine | mimics natural aggregation state of Aβ soluble oligomers or protofibrils, induces antibodies that selectively bind to toxic Aβ oligomers, promoting their clearance and neutralizing their neurotoxic effects[27-29] | ALZ-101 [30] |
